# Epistasis between *FLG* and *IL4R* Genes on the Risk of Allergic Sensitization: Results from Two Population-Based Birth Cohort Studies

**DOI:** 10.1038/s41598-018-21459-x

**Published:** 2018-02-19

**Authors:** Ali H. Ziyab, Jenny Hankinson, Susan Ewart, Eric Schauberger, Kamilla Kopec-Harding, Hongmei Zhang, Adnan Custovic, Hasan Arshad, Angela Simpson, Wilfried J. Karmaus

**Affiliations:** 1 Department of Community Medicine and Behavioral Sciences, Faculty of Medicine, Kuwait University, Kuwait City, Kuwait; 2 Division of Infection, Immunity and Respiratory Medicine, Manchester Academic Health Science Centre, The University of Manchester, Manchester University NHS Foundation Trust, Manchester, UK; 3 College of Veterinary Medicine, Michigan State University, East Lansing, Michigan USA; 4 Division of Allergy and Immunology, Cincinnati Children’s Hospital, Cincinnati, OH USA; 5 Centre for Respiratory Medicine and Allergy, Institute of Inflammation and Repair, University of Manchester, University Hospital of South Manchester NHS Foundation Trust, Manchester, UK; 6 Division of Epidemiology, Biostatistics and Environmental Health, School of Public Health, University of Memphis, Memphis, TN USA; 7 Department of Paediatrics, Imperial College London, London, UK; 8 David Hide Asthma and Allergy Research Centre, St Mary’s Hospital, Isle of Wight, UK; 9 Clinical and Experimental Sciences Academic Unit, Faculty of Medicine, University of Southampton, Southampton, UK

## Abstract

Immune-specific genes as well as genes responsible for the formation and integrity of the epidermal barrier have been implicated in the pathogeneses of allergic sensitization. This study sought to determine whether an epistatic effect (gene-gene interaction) between genetic variants within interleukin 4 receptor (*IL4R*) and filaggrin (*FLG*) genes predispose to the development of allergic sensitization. Data from two birth cohort studies were analyzed, namely the Isle of Wight (IOW; n = 1,456) and the Manchester Asthma and Allergy Study (MAAS; n = 1,058). In the IOW study, one interaction term (*IL4R* rs3024676 × *FLG* variants) showed statistical significance (interaction term: *P* = 0.003). To illustrate the observed epistasis, stratified analyses were performed, which showed that *FLG* variants were associated with allergic sensitization only among *IL4R* rs3024676 homozygotes (OR, 1.97; 95% CI, 1.27–3.05; *P* = 0.003). In contrast, *FLG* variants effect was masked among *IL4R* rs3024676 heterozygotes (OR, 0.53; 95% CI, 0.22–1.32; *P* = 0.175). Similar results were demonstrated in the MAAS study. Epistasis between immune (*IL4R*) and skin (*FLG*) regulatory genes exist in the pathogenesis of allergic sensitization. Hence, genetic susceptibility towards defective epidermal barrier and deviated immune responses could work together in the development of allergic sensitization.

## Introduction

Allergic sensitization, defined as the propensity to produce immunoglobulin E (IgE) antibodies in response to environmental and food antigens, affects up to 50% of the general population and is a common thread linking and predisposing to different manifestations of allergic diseases, including asthma, eczema, and rhinitis^1,2^. Hence, in order to understand the immunologic dysregulation that characterizes allergic diseases, it is crucial to study the etiology of allergic sensitization. Genetic susceptibility, in addition to environmental and immunologic factors, has been implicated in the pathogenesis of allergic sensitization.

Genome-wide association studies (GWAS) and candidate-gene studies have identified genetic variants across different genes that relate to the development of allergic sensitization^3–7^. Genetic variants that regulate immune responses such as interleukin 4 receptor (*IL4R*), through which IL-4 and IL-13 cytokines exert their biological regulation of IgE production, have been investigated in relation to allergic sensitization due to their relevant function in allergic inflammatory process^8^. It has also been suggested that loss-of-function variants within the filaggrin gene (*FLG*), leading to impaired skin barrier that is characterized by enhanced allergen penetration, increase the risk of allergic sensitization^9,10^. Hence, immune specific genes as well as genes responsible for the formation of functional skin barrier may both be involved in the pathogenesis of allergic sensitization. Taking a hypothesis-driven candidate-gene approach, we aimed to determine whether genetic variants within *IL4R* and *FLG* genes have an epistatic effect (interact) on allergic sensitization.

Thus far, genetic variants identified in GWAS have yielded modest effect sizes (i.e., risk/odds ratios of 1.1 to 1.5) that explain small proportions of the observed phenotypic variations of complex diseases^11^. One explanation is that the GWAS considers the effect of single nucleotide polymorphisms (SNPs) in isolation, while genes may require interaction with other genes in causal pathway for clinical manifestation. Thus, gene-gene interactions (epistasis), a setting in which the effect of one genetic variant on a phenotype depends on the genotype of another variant elsewhere in the genome, have contributed to improve results of genetic association studies^12,13^. Epistasis between immune related genes has been investigated previously in relation to the development of allergic sensitization^8,14–16^; however, epistasis between immune regulatory genes (e.g., *IL4R*) and genes that regulate the formation and function of the skin barrier (e.g., *FLG*) has not been previously reported.

We hypothesized that genetic predisposition towards defective epidermal barrier (i.e., *FLG* loss-of-function variants) in combination with genetic susceptibility for immune dysregulation (i.e., *IL4R* variants) is associated with increased risk of developing allergic sensitization. The rationale is that exposing immune-related cells residing in the epidermis to allergenic substances that easily penetrate the defective epidermal barrier may increase the risk of allergic sensitization in genetically susceptible individuals. Therefore, exploring the epistatic effect between two functionally different genes that possibly work in concert can provide new insights into the etiology of allergic sensitization. To this end, we analyzed data from two population-based birth cohort studies, namely the Isle of Wight birth cohort (IOW; discovery study) and the Manchester Asthma and Allergy Study (MAAS; replication study).

## Methods

### Isle of Wight birth cohort study – Discovery population

#### Study population

An unselected population-based birth cohort was recruited from all births (n = 1,536) occurring between January 1989 and February 1990 on the Isle of Wight, UK, to prospectively study the natural history and etiology of allergic conditions. After exclusion of adoptions, perinatal deaths, and refusal for follow-up, written informed consent was obtained from parents to enroll 1,456 (95%) newborns, with follow-up assessments conducted at 1, 2, 4, 10, and 18 years of age. Ethics approvals were obtained from the Isle of Wight Local Research Ethics Committee (now named the National Research Ethics Service, NRES Committee South Central – Southampton B) at recruitment and for the subsequent follow-ups (06/Q1701/34). The study was conducted in accordance with principles and guidelines of the Declaration of Helsinki for medical research involving human subjects.

#### Skin prick test

Skin prick tests (SPTs) were performed on most children attending the research center at ages 4 (n = 982), 10 (n = 1,036), and 18 (n = 853) years to determine their allergic sensitization status. A standard battery of common allergens (ALK-Albello, Horsholm, Denmark) was tested. Inhalant allergens tested were house dust mite, cat, dog, *Alternaria alternata*, *Cladosporium herbarium*, grass pollen mix, and tree pollen mix. Food allergens tested were cows’ milk, soya, hens’ egg, peanut and cod. Positive and negative controls were included. Allergic sensitization was defined by having a positive SPT to at least one allergen test with mean wheal diameter of 3 mm greater than the negative control.

#### Genotyping

Genomic DNA was extracted from blood or saliva samples of study participants (n = 1,211). *FLG* variants R501X, 2282del4, and S3247X were genotyped using GoldenGate Genotyping Assays on the BeadXpressVeracode platform (Illumina, Inc, SanDiego, CA, USA) per Illumina’s protocol. Individuals carrying the minor allele for at least one of the *FLG* variants R501X, 2282del4, or S3247X were classified as having filaggrin haploinsufficiency. Detailed information on *FLG* genotyping is provided by Ziyab *et al*.^17^. Single nucleotide polymorphisms (SNPs; n = 13; rs8832, rs1110470, rs1805011, rs1805012, rs2057768, rs3024604, rs3024622, rs3024676, rs3024685, rs4787423, rs6498012, rs12102586, and rs16976728) spanning the genomic region of *IL4R* gene were selected for genotyping based on a tagging scheme aiming to capture common/functional genetic variants that are related to allergic conditions across the *IL4R* gene^18^ (see Supplementary Methods online for detailed information on genotyping).

### Manchester Asthma and Allergy study – Replication population

#### Study population

MAAS is a population-based birth cohort study described in detail elsewhere (registration: ICRCTN72673620)^19^. Subjects were recruited prenatally from 1995 to 1997, and a total of 1,184 participants were born into the study, of whom 1,058 Caucasians were included in this analysis. Subjects were followed prospectively and attended follow-up clinics with a clinical assessment including sample collection, questionnaire data collection, and lung function assessment. The study was approved by the North West – Greater Manchester East Research Ethics Committee; parents gave written informed consent, and children gave assent if appropriate. The study was conducted in accordance with principles and guidelines of the Declaration of Helsinki for medical research involving human subjects.

#### Skin prick test

We ascertained sensitization to allergens by skin prick testing at ages 5 years (n = 827), 8 years (n = 818), and 11 years (n = 728) to a panel of allergens (house dust mite [Dermatophagoides pteronyssinus], cat, dog, mixed grasses, mixed trees, mixed molds, peanut, milk, and egg [Bayer, Elkahrt, IN, USA]). Allergic sensitization was defined as having a positive SPT to at least one allergen with mean wheal diameter of 3 mm greater than the negative control.

#### Genotyping

*FLG* genotyping was performed with probes and primers as previously described^20^. Genotyping for R501X and S3247X loss-of-function mutations was performed with a TaqMan-based allelic discrimination assay (Applied Biosystems, Cheshire, UK). Mutation 2282del4 was genotyped by sizing of a fluorescently labelled PCR fragment on a 3100 DNA Sequencer (ABI). Data were analyzed as combined carriage of a *FLG* null allele; that is, if a child carried 1 or more of the 3 genetic variations, he or she was considered filaggrin haploinsufficiency. Genotyping for *IL4R* SNP rs3024676 was also carried out using standard TaqMan assay conditions (assay ID: C__22272514_10).

### Statistical analysis

In the IOW study, deviation from Hardy-Weinberg Equilibrium (HWE) was tested for each of the 13 genotyped *IL4R* SNPs using goodness-of-fit χ^2^ tests (see Supplementary Table S1 online) and estimates of linkage disequilibrium (LD) between SNPs were calculated using *D*ʹ and *r*^2^ measures (see Supplementary Fig. S1). SNPs that deviated from HWE (*P* < 0.05) were excluded from this analysis (1 SNP; rs3024622). Since allergic sensitization (the outcome variable) was repeatedly measured at ages 4, 10, and 18 years, generalized linear statistical models were applied with parameters estimated via generalized estimating equations (GEE) to account for the correlated observations and the within-child effect^21^. For each *IL4R* SNP, the best fitting genetic model (additive, dominant, recessive, or heterosis; see Supplementary Methods online) was determined based on the quasi-likelihood under the independence model criterion (QIC), which is used to assess the goodness-of-fit in GEEs^22^. In this selection step, GEE models included the main effect of the respective *IL4R* SNP (under the respective genetic model) as the independent variable (age and sex were included as potential confounders) and the repeated measures of allergic sensitization were considered as the outcome variable; for each *IL4R* SNP, the genetic model with the lowest QIC value was selected as the best fitting model. Using the best fitting genetic model for each of *IL4R* SNPs, the statistical significance of interaction terms between *IL4R* SNPs and *FLG* variants were tested in logistic regression models (total of 12 tests). To adjust for multiple testing, we controlled the false discovery rate (FDR) to obtain multiple-testing corrected p-values associated with the interaction terms^23^. In the replication population (MAAS cohort), we only tested statistical interactions between *IL4R* SNPs with *FLG* variants that demonstrated statistical significance (FDR *P* < 0.05) in the discovery study. Age and sex were included in all generalized linear equations as potential confounders. The procedure GENMOD in SAS 9.4 (SAS, Gary, NC, USA) was applied. Odds ratios (ORs) and their associated 95% confidence intervals (CIs) were estimated.

### Data availability

The datasets analyzed during the current study are available from the corresponding author on reasonable request.

## Results

### Description of study populations

In both studies allergic sensitization increased in prevalence with age. For example, among the IOW study participants, SPT positivity was observed in 19.7% at 4 years, 26.9% at 10 years, and 41.4% at 18 years of age (Table 1). In the MAAS cohort, the proportion of positive SPTs increased from 29.0% to 35.2% between ages 5 and 11 years. The combined proportion of carrying the minor allele(s) of *FLG* variants R501X, 2282del4, and S3247X was 10.3% in the IOW cohort and 9.9% in the MAAS cohort (Table 1).

**Table 1:** Characteristics of the IOW and MAAS (Caucasians only) cohort studies participants.

Characteristic	IOW cohort % (n/total)	MAAS cohort % (n/total)
**Sex**		
Male	51.2 (745/1456)	54.5 (577/1058)
Female	48.8 (711/1456)	45.5 (481/1058)
**Allergic sensitization at age**		
4 years	19.6 (192/982)	—
5 years	—	29.0 (240/827)
8 years	—	32.8 (268/818)
10 years	26.9 (279/1036)	—
11 years	—	35.2 (256/728)
18 years	41.4 (353/853)	—
***IL4R*** **rs3024676**		
AA	2.3 (26/1149)	3.3 (30/907)
AC	29.1 (335/1149)	29.2 (265/907)
CC	68.6 (788/1149)	67.5 (612/907)
MAF (allele A)	0.17	0.18
HWE *P* value	0.199	0.841
***FLG*** **variants**		
R501X	4.1 (47/1161)	4.6 (40/870)
2282del4	4.6 (54/1168)	4.5 (37/828)
S3247X	1.6 (18/1165)	0.8 (7/909)
***FLG*** **haploinsufficiency** ^*^	10.3 (118/1150)	9.9 (80/809)

MAF: Minor allele frequency; HWE: Hardy-Weinberg equilibrium.

^*^Analyses were conducted using the combined carriage of a *FLG* null allele; that is, if a child carried 1 or more of the *FLG* variants R501X, 2282del4, or S3247X, he or she was classified as having filaggrin haploinsufficiency.

### Selecting best-fitting genetic model and screening for epistasis

In the IOW cohort study (discovery study), the best-fitting genetic model (additive, dominant, recessive, or heterosis) was selected for each of the twelve *IL4R* SNPs based on the smallest QIC value calculated for generalized linear models fitted using GEE (see Supplementary Table S2); these models tested the association between *IL4R* SNPs under the different genetic models with the repeated measurements of allergic sensitization. Subsequently, twelve interaction terms (*IL4R* SNP × *FLG* variants) were evaluated using logistic regression equations (Table 2). After correcting for multiple testing, one interaction term (*IL4R* rs3024676 × *FLG* variants) demonstrated statistical significance (interaction term: *P* = 0.003, FDR corrected *P* = 0.036, Table 2).

**Table 2:** Evaluating statistical interaction effects (epistasis) between *IL4R* SNPs and *FLG* variants on the risk of allergic sensitization: results from IOW cohort study via longitudinal analyses.

*IL4R* SNPs	Genetic Model^†^	Genotype^†^	Interaction term (*IL4R* × *FLG*)^‡^
			*P* value
rs8832	Additive	GG/AG/AA	0.791
rs1110470	Additive	GG/AG/AA	0.388
rs1805011	Heterosis	AA/CC vs. AC	0.389
rs1805012	Dominant	GG/AG vs. AA	0.929
rs2057768	Additive	GG/AG/AA	0.412
rs3024604	Heterosis	AA/GG vs. AG	0.891
rs3024676	Heterosis	AA/CC vs. AC	**0.003** ^*^
rs3024685	Additive	GG/AG/AA	0.703
rs4787423	Recessive	GG vs. AA/AG	0.649
rs6498012	Additive	GG/CG/CC	0.878
rs12102586	Recessive	AA vs. AG/GG	0.845
rs16976728	Dominant	AA/AG vs. GG	0.809

SNP: Single nucleotide polymorphism.

^*^FDR corrected *P* value = 0.036.

^†^For each *IL4R* SNP the best fitting genetic risk model (dominant, recessive, heterosis, or additive) was selected as detailed in the ‘Methods’ section. Under the additive model, the three genotypes were coded and entered in the regression model in a dosage-effect manner (from smaller to larger effect). For instance, the best fitting genetic risk model for SNP rs8832 is ‘additive’ model, meaning that the risk of allergic sensitization increases gradually from least risk among those with GG genotype to moderate risk among those with AG genotype to highest risk among those with AA genotype. In the ‘heterosis’ model we collapsed the two homozygote genotypes together and compare their risk to the heterozygote risk. For example, the best fitting genetic risk model for SNP rs1805011 is ‘heterosis’ model, in which the risk of homozygote genotypes (AA/CC) was compared to the risk of heterozygote genotype (AC). For SNP rs1805012, in the dominant model we compared the risk of variant homozygote and heterozygote (GG/AG) genotypes to the risk of wild-type homozygote (AA) genotype. For SNP rs4787423, in the recessive model we compared the risk of variant homozygote (GG) genotype to the risk of wild-type homozygote and heterozygote (AA/AG) genotypes.

^‡^Interaction term refers to the product term that we included in the regression model to test for the presence of multiplicative statistical interaction between *FLG* variants and *IL4R* SNPs. Since there are 12 *IL4R* SNPs, we tested 12 interaction terms (*IL4R* SNP × *FLG* variants), while using the best fitting genetic risk model for each *IL4R* SNP.

### Illustrating the epistatic effect between *FLG* and *IL4R* – IOW cohort study

First, we assessed the association of *FLG* variants and *IL4R* rs3024676 with allergic sensitization to determine their independent effects. *FLG* variants showed positive association with allergic sensitization based on results of repeated measurements analysis that covered the period from 4 to 18 years of age (OR, 1.47; 95% CI, 1.01–2.14; *P* = 0.046; Table 3). In contrast, the association between *IL4R* rs3024676 (heterosis genetic model: AA/CC vs. AC) and repeated measurements of allergic sensitization did not demonstrate statistical significance (OR, 1.25; 95% CI, 0.97–1.62; *P* = 0.085; Table 3). However, *IL4R* rs3024676 was associated with increased risk of allergic sensitization at age 10 years (OR, 1.56; 95% CI, 1.12–2.16; *P* = 0.008).

**Table 3:** Association of *FLG* variants and *IL4R* rs3024676 with allergic sensitization: results from IOW cohort study.

Allergic sensitization at age	*FLG* variants		*IL4R* rs3024676 genotype	
	WT	LOF	AC	AA/CC
**4 years**, % (n/total)	19.6 (147/751)	27.2 (25/92)	19.2 (46/240)	21.3 (129/605)
OR (95% CI)^*^	1.00	1.51 (0.92–2.47)	1.00	1.15 (0.79–1.68)
*P* value		0.105		0.468
**10 years**, % (n/total)	26.4 (227/859)	41.1 (39/95)	22.2 (63/284)	30.2 (201/666)
OR (95% CI)^*^	1.00	1.89 (1.22–2.93)	1.00	1.56 (1.12–2.16)
*P* value		0.004		0.008
**18 years**, % (n/total)	41.3 (293/709)	50.6 (43/85)	38.1 (86/226)	44.0 (250/568)
OR (95% CI)^*^	1.00	1.41 (0.89–2.23)	1.00	1.32 (0.96–1.81)
*P* value		0.140		0.090
**Repeated measurements**, % (k/total)^†^	28.8 (667/2319)	39.3 (107/272)	26.0 (195/750)	31.5 (580/1839)
OR (95% CI)	1.00	1.47 (1.01–2.14)	1.00	1.25 (0.97–1.62)
*P* value		0.046		0.085

*FLG* WT: *FLG* wild-type genotype; *FLG* LOF: *FLG* loss-of-function genotype; k = number of repeated measurements.

^*^Association adjusted for sex.

^†^Association adjusted for sex and age at follow-up.

Next, to illustrate the possible epistasis (*IL4R* rs3024676 × *FLG* variants), the association between *FLG* variants with allergic sensitization was stratified by *IL4R* rs3024676 genotypes. The stratified analysis showed that *FLG* variants statistically significantly associated with increased risk of allergic sensitization among *IL4R* rs3024676 homozygotes (repeated measurements analysis: OR, 1.97; 95% CI, 1.27–3.05; *P* = 0.003; Table 4). In contrast, *FLG* variants did not pose any increased risk for allergic sensitization among *IL4R* rs3024676 heterozygotes (repeated measurements analysis: OR, 0.53; 95% CI, 0.22–1.32; *P* = 0.175; Table 4). Hence, indicating that *IL4R* rs3024676 is a possible effect modifier of the association between *FLG* variants with allergic sensitization.

**Table 4:** Association of *FLG* variants with allergic sensitization stratified by *IL4R* rs3024676 genotypes: results from IOW cohort study.

Allergic sensitization at age	*IL4R* rs3024676 Homozygous (AA/CC)		*IL4R* rs3024676 Heterozygous (AC)	
	*FLG* WT	*FLG* LOF	*FLG* WT	*FLG* LOF
**4 years**, % (n/total)	20.1 (107/532)	30.3 (20/66)	18.3 (39/213)	21.7 (5/23)
OR (95% CI)^*^	1.00	1.71 (0.97–3.01)	1.00	1.20 (0.42–3.44)
*P* value		0.064		0.738
**10 years**, % (n/total)	28.2 (168/595)	50.0 (31/62)	22.8 (57/250)	20.7 (6/29)
OR (95% CI)^*^	1.00	2.55 (1.50–4.35)	1.00	0.86 (0.33–2.23)
*P* value		< 0.001		0.762
**18 years**, % (n/total)	42.3 (212/501)	60.7 (37/61)	40.5 (81/200)	19.1 (4/21)
OR (95% CI)^*^	1.00	2.07 (1.20–3.59)	1.00	0.32 (0.10–0.99)
*P* value		0.009		0.048
**Repeated measurements**, % (k/total)^†^	29.9 (487/1628)	46.6 (88/189)	26.7 (177/663)	20.6 (15/73)
OR (95% CI)	1.00	1.97 (1.27–3.05)	1.00	0.53 (0.22–1.32)
*P* value		0.003		0.175

*FLG* WT: *FLG* wild-type genotype; *FLG* LOF: *FLG* loss-of-function genotype; k = number of repeated measurements.

^*^Association adjusted for sex.

^†^Association adjusted for sex and age at follow-up.

### Replicating the epistasis between *FLG* and *IL4R* – MAAS cohort

In the MAAS cohort (replication study), *FLG* variants demonstrated a statistically significant association with the repeated measurements of allergic sensitization status (OR, 1.73; 95% CI, 1.1.3–2.63; *P* = 0.011; Table 5). Similar to findings from the IOW cohort study, there was no association between *IL4R* rs3024676 and allergic sensitization (repeated measurements analysis: OR, 0.87; 95% CI, 0.65–1.15; *P* = 0.328; Table 5). When testing for statistical interaction on a multiplicative scale, the interaction term (*IL4R* rs3024676 × *FLG* variants) did not demonstrate statistical significance (interaction term: *P* = 0.536) in the MAAS cohort. However, similar to findings from the IOW study, in the analysis stratified by *IL4R* rs3024676 genotype, *FLG* variants were associated with increased risk of allergic sensitization among *IL4R* rs3024676 homozygotes (repeated measurements analysis: OR, 1.93; 95% CI, 1.12–3.31; *P* = 0.017; Table 6). And there was no association between *FLG* variants and allergic sensitization among *IL4R* rs3024676 heterozygotes (repeated measurements analysis: OR, 1.44; 95% CI, 0.66–3.12; *P* = 0.360; Table 6).

**Table 5:** Association of *FLG* variants and *IL4R* rs3024676 with allergic sensitization: results from MAAS cohort study

Allergic sensitization at age	*FLG* variants		*IL4R* rs3024676 genotype	
	WT	LOF	AC	AA/CC
**5 years**, % (n/total)	28.2 (187/662)	44.8 (30/67)	29.8 (67/225)	28.9 (157/543)
OR (95% CI)^*^	1.00	1.97 (1.18–3.30)	1.00	0.97 (0.69–1.37)
*P* value		0.010		0.871
**8 years**, % (n/total)	31.3 (202/646)	48.6 (36/74)	35.0 (76/217)	31.1 (169/543)
OR (95% CI)^*^	1.00	2.05 (1.25–3.35)	1.00	0.84 (0.60–1.18)
*P* value		0.004		0.321
**11 years**, % (n/total)	34.8 (202/581)	43.8 (28/64)	39.6 (76/192)	32.5 (159/489)
OR (95% CI)^*^	1.00	1.47 (0.86–2.49)	1.00	0.74 (0.52–1.04)
*P* value		0.155		0.087
**Repeated measurements**, % (k/total)^†^	31.3 (591/1889)	45.9 (94/205)	34.5 (219/634)	30.8 (485/1575)
OR (95% CI)	1.00	1.73 (1.13–2.63)	1.00	0.87 (0.65–1.15)
*P* value		0.011		0.328

*FLG* WT: *FLG* wild-type genotype; *FLG* LOF: *FLG* loss-of-function genotype; k = number of repeated measurements.

^*^Association adjusted for sex.

^†^Association adjusted for sex and age at follow-up.

**Table 6:** Association of *FLG* variants with allergic sensitization stratified by *IL4R* rs3024676 genotypes: results from MAAS cohort study.

Allergic sensitization at age	*IL4R* rs3024676 Homozygous (AA/CC)		*IL4R* rs3024676 Heterozygous (AC)	
	*FLG* WT	*FLG* LOF	*FLG* WT	*FLG* LOF
**5 years**, % (n/total)	28.2 (124/439)	46.2 (18/39)	28.6 (52/182)	42.9 (9/21)
OR (95% CI)^*^	1.00	2.11 (1.08–4.11)	1.00	1.74 (0.68–4.45)
*P* value		0.028		0.249
**8 years**, % (n/total)	29.4 (128/436)	52.2 (24/46)	33.9 (58/171)	45.5 (10/22)
OR (95% CI)^*^	1.00	2.58 (1.39–4.81)	1.00	1.60 (0.65–3.94)
*P* value		0.003		0.307
**11 years**, % (n/total)	31.8 (125/393)	45.0 (18/40)	40.0 (62/155)	41.2 (7/17)
OR (95% CI)^*^	1.00	1.71 (0.88–3.34)	1.00	1.08 (0.39–2.99)
*P* value		0.115		0.889
**Repeated measurements**, % (k/total)^†^	29.7 (377/1268)	48.0 (60/125)	33.9 (172/508)	43.3 (26/60)
OR (95% CI)	1.00	1.93 (1.12–3.31)	1.00	1.44 (0.66–3.12)
*P* value		0.017		0.360

*FLG* WT: *FLG* wild-type genotype; *FLG* LOF: *FLG* loss-of-function genotype; k = number of repeated measurements.

^*^Association adjusted for sex.

^†^Association adjusted for sex and age at follow-up.

## Discussion

The current study demonstrates an epistatic (gene-gene interaction) effect between genetic variants in the *FLG* and *IL4R* genes on the development of allergic sensitization in two independent population-based birth cohort studies. In the IOW and MAAS study cohorts, *FLG* variants increased the risk of allergic sensitization in the total study samples; however, in the analysis stratified by *IL4R* rs3024676 SNP genotypes, *FLG* variants only increased the risk of allergic sensitization among participants who carry the homozygous genotypes (i.e., AA or CC) of *IL4R* rs3024676 SNP. Subsequently, the effect of *FLG* variants on the risk of allergic sensitization was masked among those with the heterozygous genotype (i.e., AC) of *IL4R* rs3024676 SNP. Hence, results of this report indicate that *IL4R* rs3024676 SNP is a possible effect modifier of the association between *FLG* variants and allergic sensitization; suggesting an interplay between immune-specific (*IL4R*) and skin-specific (*FLG*) genes in the pathogenesis of allergic sensitization.

The role of *IL4R* genetic variants in the development of allergic disorders and sensitization is well documented in the scientific literature^8,24,25^. Briefly, both IL-4 and IL-13 cytokines can activate IL4R, which subsequently activates the Janus kinase/signal transducer and activator of transcription (JAK/STAT) signaling pathways leading to the synthesis of IgE^26^. Hence, IL4R plays a central as well as a critical role in the regulation of IgE-mediated allergic inflammation. On the other hand, the role of epidermal barrier defects in the development of allergic manifestation has been further highlighted after the discovery of *FLG* loss-of-function variants^20^. The mechanically impaired epidermal barrier allows allergen penetration and facilitates uptake of allergens by antigen-presenting cells residing in the skin (e.g., Langerhans cells, dendritic cells), which subsequently induces cutaneous allergen sensitization^27,28^. Therefore, genetic susceptibility towards defective epidermal barrier and deviated immune responses could work together in the development of allergic sensitization. The finding of possible epistasis between two functionally different genes that work in concert in the development of allergic disorders and sensitization highlight the importance of considering epistasis (gene-gene interaction) in genetic association studies. Although the current report focuses on gene-gene interactions, considering gene-environment interactions is as important and should concurrently be incorporated in future studies to determine whether risks associated with environmental exposures are the same for people with different genetic susceptibilities.

Prior investigations have shown that epistasis between immune-specific genes contributes to the development of allergic sensitization (influence serum IgE levels). For instance, combining variants in *IL4* (rs2243250), *IL13* (rs1800925), *IL4R* (rs1805010), and *STAT6* (rs324011) genes was associated with a 10.8-fold increased risk of having high serum IgE levels (i.e., IgE >457 IU/mL [>90th percentile])^16^. Moreover, a gene-gene interaction between *IL13* and *CCL17* (chemokine (C-C motif) ligand 17, also known as *TARC*: thymus and activation-regulated chemokine) variants increased the risk of having at least one positive allergen-specific IgE test response by 3.9-fold^29^.

The current study further confirmed the previously noted association between *FLG* variants and allergic sensitization^10^ and added that this association is modified by *IL4R* rs3024676 SNP. The observed epistasis suggests that *FLG* variants exert their adverse effect only among individuals carrying the homozygous genotypes of *IL4R* rs3024676 SNP. Surprisingly, *FLG* variants posed no increased risk of allergic sensitization if individuals carried the heterozygous genotype of *IL4R* rs3024676 SNP. Hence, indicating that carrying the two alleles (A and C) of *IL4R* rs3024676 SNP masked the effect of *FLG* variants. In this sense, having a heterozygous genotype of *IL4R* rs3024676 SNP is advantageous for individuals carrying *FLG* variants. The ‘heterozygote advantage’ (heterosis) phenomenon, whereby carrying two different versions of the gene (heterozygous genotype) is more advantageous than having two copies of the same allele (homozygous genotype), has long been recognized in animal and plant genetics, but largely overlooked in human genetic studies^30,31^. Although rarely investigated, heterosis is believed to be a common phenomenon in humans and not accounting for the heterotic effect hinders the success of genetic linkage and association studies^32,33^. A classic example of ‘heterozygote advantage’ is demonstrated through the elevated tolerance/resistance to malaria infection among individuals who are heterozygous for sickle cell anemia causing-allele as compared to those who are homozygous at this locus^34^.

To corroborate the observed differential effect of the heterozygous genotype (heterosis model) of *IL4R* rs3024676, we assessed associations between genotypes of *IL4R* rs3024676 SNP with IL-4 cytokine levels measured in serum of subset of IOW study participants aged 10 years. Results of this proof-of-concept analysis showed that serum levels of IL-4 cytokine varies cross genotypes of *IL4R* rs3024676 with higher levels observed among participants carrying the heterozygous genotype compared to those carrying the homozygous genotypes (*P* = 0.10; see Supplementary Table S3). Moreover, since DNA methylation was measured in blood samples of a subset of IOW study participants, we further investigated whether DNA methylation levels at CpG (cytosine-phosphate-guanine) sites within the *IL4R* gene are associated with genotypes of *IL4R* rs3024676 SNP. Results of this analysis showed that levels of DNA methylation of *IL4R* CpG site to be lower among participants with the heterozygous genotype of *IL4R* rs3024676 SNP compared to participants with homozygous genotypes (*P* = 0.001; see Supplementary Table S4). Hence, the previous findings strengthen the plausibility of a heterosis model. However, further investigations from a genetic point of view, e.g., through experiments, are needed to conform the mechanistic plausibility of the heterosis model.

The longitudinal design of both the IOW and MAAS cohort studies that prospectively ascertained allergic sensitization status through repeated skin prick testing is a major strength of the current study. Applying repeated measurements analysis helped in minimizing the effect of misclassification, if any is present, on the results of this report. Moreover, in both studies the proportions of children who participated in SPTs remained reasonably high throughout the follow-ups. In the IOW cohort study, SPT was performed on 982 (80%), 1036 (75%), and 853 (65%) of those who participated in the 4-, 10-, and 18-years follow-ups, respectively. In a previous report we demonstrated that there is no indication of selection bias in terms of participants undergoing skin prick testing at each assessment in regard to various phenotypes^35^. In the MAAS cohort study, information on allergic sensitization status was available for 827 (78%), 818 (77%), and 728 (69%) participants at ages 5, 8, and 11 years, respectively. The slight discrepancies in the prevalence of allergic sensitization between the IOW and MAAS cohort studies can be explained by the different timing (age of participants) of ascertainment. In the stratified analyses by *IL4R* rs3024676 genotypes, the association of *FLG* variants with allergic sensitization among *IL4R* rs3024676 homozygotes showed agreement in regard to the direction of the effect measure and statistical significance in both studies (IOW: OR, 1.97, *P* = 0.003, Table 4; MAAS: OR, 1.93, *P* = 0.017, Table 6). In contrast, among *IL4R* rs3024676 heterozygotes the direction of the effect measure disagreed across the two studies (IOW: OR, 0.53, *P* = 0.175, Table 4; MAAS: OR, 1.44, *P* = 0.360, Table 6); however, this inconsistency has no impact on our findings/conclusions due to the lack of statistical significance in either study and the consistent/strong combined effect of *IL4R* AA/CC and *FLG* variants on the risk of allergic sensitization in both studies (see Supplementary Fig. S2). On a multiplicative scale, the statistical significance of the interaction term (*IL4R* rs3024676 × *FLG* variants) that was observed in the IOW study was not replicated in the MAAS cohort. However, observing an association between *FLG* variants and allergic sensitization among *IL4R* rs3024676 homozygotes in both studies and the absence of such association among *IL4R* rs3024676 heterozygotes indicates that *IL4R* rs3024676 SNP is a potential effect modifier and that the findings were consistent with regard to their directionality a statistical significance in both studies among *IL4R* rs3024676 homozygotes. Although the signal for a statistical epistasis was moderate and marginally replicated, the functional evidence, i.e. differential serum levels of IL-4 cytokine and DNA methylation levels at CpG sites within the *IL4R* gene among participants with heterozygous genotype of *IL4R* rs3024676 SNP compared to participants with homozygous genotypes (see Supplementary Table S3 and Table S4), further strengthens the evidence of possible epistatic effect and the plausibility of a heterosis risk model. Future studies are needed to corroborate our findings.

*IL4R* rs3024676 SNP is an intron polymorphism, thus its epistatic effect may be due to its being in linkage disequilibrium with a functional variant that is responsible for the observed epistatic effect. However, a prior investigation has shown that *IL4R* rs3024676 SNP is part of an interaction of six genes affecting pediatric asthma^36^. Another drawback is the use of OR as effect measure, which can overestimate the risk ratio when the outcome is common (i.e., >10%)^37^. To assess the possible overestimation, using IOW cohort study data, we estimated the risk ratio for the association between *FLG* variants and allergic sensitization among those who carry the homozygous genotypes of *IL4R* rs3024676 SNP; the risk ratio estimate is 1.40 (95% CI, 1.13–1.75; *P* = 0.003) and the reported OR is 1.97 (95% CI, 1.27–3.05; *P* = 0.003). In both approaches the statistical significance did not change, but the magnitude of the effect was influenced by the choice of the effect measure. In the current study we estimated ORs using logistic regression due to its availability to both research groups.

In conclusion, the current study demonstrates, for the first time, that epistatic effect (gene-gene interaction) between immune (*IL4R*) and skin (*FLG*) regulatory genes exist in the pathogenesis of allergic sensitization in two independent epidemiological studies. *FLG* variants predisposed to increased risk of allergic sensitization among those who carry the homozygous genotypes of *IL4R* rs3024676 SNP; whereas, *FLG* variants effect was masked among those who are heterozygous for *IL4R* rs3024676 SNP. Based on results of this report, it is plausible that epidermal barrier and immune regulatory genes act synergistically (or additively) in the development of allergic sensitization. Hence, the observed epistasis provides insight on possible mechanisms underlying the development of allergic sensitization.

## Electronic supplementary material

Supplementary Information
