# Supplementary material for: Epistasis between FLG and IL4R Genes on the Risk of Allergic Sensitization: Results from Two Population-Based Birth Cohort Studies
Source: Sci Rep. 2018 Feb 19;8:3221. doi: 10.1038/s41598-018-21459-x (PMC5818621; doi:10.1038/s41598-018-21459-x)
Supplement: Supplementary file 1 — Supplementary Information [file 41598_2018_21459_MOESM1_ESM.pdf]

Supplementary Information

Manuscript title: Epistasis between *FLG* and *IL4R* Genes on the Risk of Allergic Sensitization: Results from Two Population-Based Birth Cohort Studies

Authors: Ali H. Ziyab*, Jenny Hankinson, Susan Ewart, Eric Schauberger, Kamilla Kopec-Harding, Hongmei Zhang, Adnan Custovic, Hasan Arshad, Angela Simpson, Wilfried J. Karmaus

*** Corresponding author:**

Ali H. Ziyab, PhD

Departments of Community Medicine and Behavioral Sciences, Faculty of Medicine, Kuwait University, Kuwait.

Tel: (+965) 24636545

Fax: (+965) 25338948

Email: aziyab@hsc.edu.kw

Supplementary Methods

Genotyping – Isle of Wight birth cohort study

DNA was extracted from blood or saliva samples from cohort subjects (n = 1,211). *FLG* variants R501X, 2282del4, and S3247X were selected for genotyping. DNA samples were interrogated using GoldenGate Genotyping Assays (Illumina, Inc, SanDiego, CA) on the BeadXpressVeracode platform (Illumina, Inc, SanDiego, CA) per Illumina's protocol. In brief, samples were fragmented and hybridized to the pool of allele-specific primer sets. Following an extension/ligation reaction the samples were then hybridized to the Veracode bead pool and processed on the BeadXpress reader. Data were analyzed using the genotyping module of the GenomeStudio Software package (Illumina, Inc, SanDiego, CA). DNA from each subject plus 37 replicate samples were analyzed for a total of 1,248 samples. The quality threshold for allele determination was set at a GenCall score > 0.25 (scores ≤ 0.25 were "no calls") with n = 1,227 samples (98.3%) retained for further analysis. Analysis of each locus included reclustering of genotyping data using our project data to define genotype cluster positions with additional manual reclustering to maximize both cluster separation and the 50th percentile of the distribution of the GenCall scores across all genotypes (50% GC score). Participants were classified as having *FLG* loss-of-function defect if they carry the minor allele for at least one of the following *FLG* null variants: R501X, 2282del, or S3247X.

In regard to *IL4R* SNPs, an efficient genotype tagging scheme was developed that gave priority to variants that 1) showed strong association with asthma in the Isle of Wight birth cohort, and/or 2) have been reported by others to be associated with asthma/allergy, and/or 3) have functional importance. A literature search for *IL4R* gene plus asthma and allergy was used to identify

associated variants (SNPs, indels). Functional variants included those that were non-synonymous, located in conserved DNA, and/or present in DNA regions with gene regulatory potential. Tagger implemented in Haploview 3.2 using Caucasian Hapmap data was used to develop a tagging scheme for the *IL4R* gene region, including 10 kb upstream and downstream of the gene. An r^2 value of 0.85 was the threshold for tagging and one, two, and three SNP marker combination tests were used. The result was an efficient number of genotyped variants (n = 13) that would provide the needed information to statistically support or exclude the gene in its association with asthma outcomes.

Thirteen *IL4R* SNPs were genotyped (Table S5). DNA samples were interrogated using GoldenGate Genotyping Assays (Illumina, Inc, SanDiego, CA) on the BeadXpressVeracode bead platform (Illumina, Inc, SanDiego, CA) per Illumina's protocol. Data were analyzed using the genotyping module of the GenomeStudio Software package (Illumina, Inc, SanDiego, CA). DNA from each subject plus 37 replicate samples, genotyped for control purposes, were analyzed for a total of 1,248 samples (trios were not available). The quality threshold for allele determination across samples was set at a GenCall score >0.25 with n=1,227 samples retained for further analysis. Analysis of each locus included reclustering of genotyping data using our project data to define genotype cluster positions with additional manual reclustering to maximize both cluster separation and the 50th percentile of the distribution of the GenCall scores across all genotypes (50% GC score). GenCall score is a quality metric of the Illumina GenomeStudio software that indicates the reliability of the genotypes called on SNP arrays, with scores ranging from 0.0 to 1.0. The proprietary algorithm for converting raw allele intensities into genotypes

considers angle of the clusters, dispersion of clusters, overlap between clusters, and intensity. Genotypes with lower GenCall scores are located furthest from the center of the cluster.

Genetic risk models and interaction analysis

Genetic association studies examine relationships between the presence of a genotype (genetic factors) and a phenotype (traits). A genotype, is therefore the exposure of interest in genetic epidemiology. Since there are three genotypes for each SNP (e.g., AA, AB, and BB), their analysis requires some manipulation. Assuming that one of the alleles (allele B) is associated with increased risk of disease; thus, the other allele (allele A) will be a marker for protection (baseline risk). In practice, statistical analysis of genetic associations is done assuming that the uncommon (minor) allele is the risk marker. For analysis purposes, the three genotypes are coded according to the genetic risk model of interest. Commonly explored genetic risk models are: dominant, recessive, and additive and to a lesser extent some studies consider the ‘heterosis’ (over-dominant) risk model [1-4].

The dominant, recessive, and heterosis risk models require the three genotypes to be collapsed into two levels (present/absent; see Figure 1). In order to determine whether the dominant model fits the data, the heterozygote (AB) and variant homozygote (BB) genotypes are collapsed together and assumed to be the risk group and the wild-type genotype (AA) makes the baseline/referent group. In the case of the recessive model, the assumption is that the variant allele is associated with risk when present in two copies. Hence, in analysis for the recessive model, the variant homozygote (BB) genotype is consider the risk group and compared to the referent group (AA and AB genotypes). In the heterosis (heterozygote advantage or over-

dominant) model, the interest is in heterozygosity as opposed to the two homozygote genotypes. The AB genotype make one group and homozygote genotypes (AA and BB) make the comparative category. The heterosis model is rarely explored in the human genetic association literature; however, it provides insights on genomic regions where heterozygote advantage might exists. The only difference between these models is in which genotype or collapsed genotypes they consider as the risk genotype: the dominant model considers AB and BB as risk genotypes; the recessive model considers BB as the risk genotype; and heterosis model considered AB genotype to either have higher or lower risk compared to the AA and BB genotypes.

On the other hand, in some instances, genotypes are analyzed for a gene dosage effect using the 'additive' model (Figure 1). This model may be likened to the examination of association for different grades of an exposure, such as no smoking, moderate smoking, and heavy smoking. In the additive model, all three genotypes are retained as individual exposures and represent gradually increasing levels of exposure from AA (baseline), to AB (intermediate exposure), and to BB (maximum exposure).

Given the previous brief background, we explored the dominant, recessive, heterosis, and additive genetic risk models for each of the 12 *IL4R* SNPs when assessing association with allergic sensitization (the outcome). This step was done to select the best fitting genetic risk model for each of the 12 *IL4R* SNPs. Hence, for each SNP, four genetic risk models were tests in association with allergic sensitization, and the genetic model with the lowest QIC value was selected as the best fitting model. Information related to this step is provided in Table S2, which shows the QIC values for each SNP under the different genetic risk models.

After finding the best fitting genetic risk model for each *IL4R* SNP, we tested multiplicative statistical interactions, by including a product term in regression models, between *FLG* variants (defined using dominant model as present/absent) and *IL4R* SNPs (using the best fitting model found in Table S2) on the risk of allergic sensitization. Hence, we evaluated 12 models to determine if there is statistical interaction between *FLG* variants and any of the 12 *IL4R* SNPs on the risk of allergic sensitization. The presence of multiplicative statistical interaction was assessed using the p-value associated with the interaction term (an FDR-adjusted p-value < 0.05 was considered statistically significant). Table 2 shows p-values of the 12 evaluated interaction terms. Of note, we used the term 'epistasis' to refer to multiplicative statistical interactions (non-additive effects) between genes at different loci.

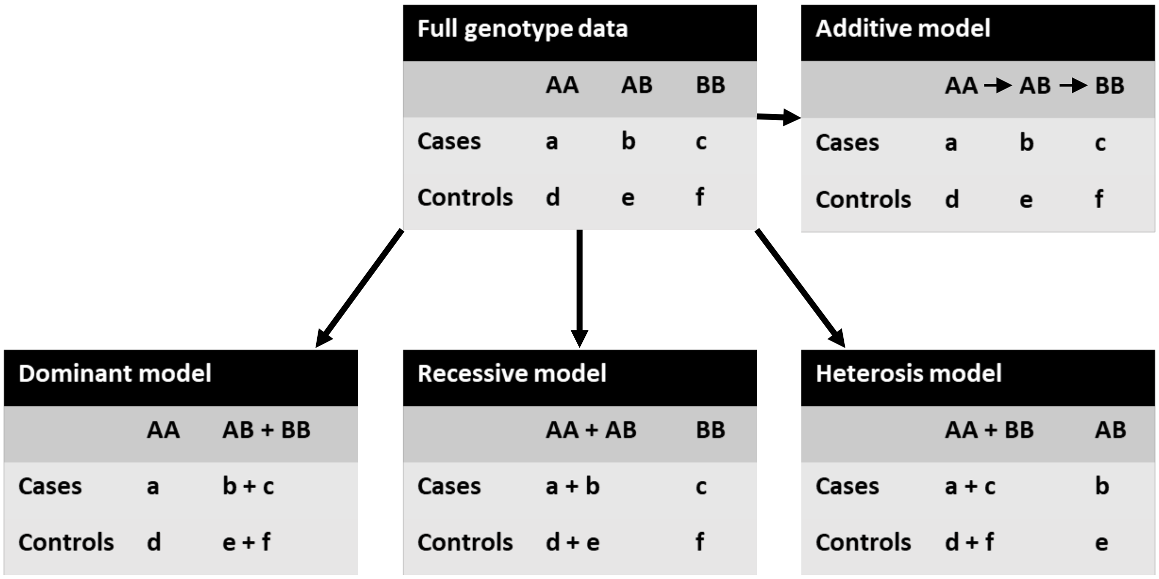

Figure 1. Illustration of genetic risk models in relation to the full genotype data.

References

1. Dorak MT, Genetic association studies: background, conduct, analysis, interpretation. New York, NY: Garland Science, Taylor & Francis Group, LLC, 2017.
2. Ziegler A, König IR, A statistical approach to genetic epidemiology. 2nd Edn. Weinheim: Wiley-VCH, 2010.
3. Lewis CM. Genetic association studies: design, analysis and interpretation. Brief Bioinform 2002;3: 146-53.
4. Horita N, Kaneko T. Genetic model selection for a case-control study and a meta-analysis. Meta Gene 2015;5: 1-8.

Table S1. Genotype and minor allele frequencies of *IL4R* single nucleotide polymorphisms: results from IOW cohort study

IL4R **SNP**	**Genotypes**	**Genotype Frequencies**	**Minor allele frequency**	**HWE *P* value**
rs8832	AA/AG/GG	254/559/333	0.47	0.532
rs1110470	AA/AG/GG	235/567/324	0.46	0.688
rs1805011	AA/AC/CC	889/244/17	0.12	0.986
rs1805012	AA/AG/GG	882/236/18	0.12	0.703
rs2057768	AA/AG/GG	100/503/555	0.30	0.398
rs3024604	AA/AG/GG	984/159/9	0.08	0.456
rs3024622	CC/CG/GG	156/466/463	0.36	0.035
rs3024676	AA/AC/CC	26/335/788	0.17	0.199
rs3024685	AA/AG/GG	406/546/196	0.41	0.602
rs4787423	AA/AG/GG	875/268/20	0.13	0.984
rs6498012	CC/CG/GG	176/564/409	0.40	0.465
rs12102586	AA/AG/GG	11/189/963	0.09	0.684
rs16976728	AA/AG/GG	182/574/401	0.41	0.361

HWE: Hardy-Weinberg equilibrium; SNP: single nucleotide polymorphism

Table S2. Selecting the best fitting genetic model based on QIC for *IL4R* single nucleotide polymorphisms: results from IOW cohort study

	**QIC for each genetic model**			
***IL4R* SNP**	**Additive**	**Dominant**	**Recessive**	**Heterosis**
rs8832	**3038.0**	3038.3	3039.3	3040.2
rs1110470	**2985.5**	2986.5	2985.6	2987.3
rs1805011	3051.6	3049.1	3055.5	**3047.3**
rs1805012	3008.0	**3006.8**	3015.4	3007.0
rs2057768	**3075.8**	3075.9	3076.4	3076.7
rs3024604	3058.1	3057.9	3059.1	**3057.7**
rs3024676	3053.7	3050.4	3053.9	**3046.9**
rs3024685	**3031.4**	3035.2	3033.3	3040.6
rs4787423	3088.0	3087.9	**3087.2**	3087.8
rs6498012	**3047.2**	3047.5	3051.3	3052.0
rs12102586	3082.3	3082.7	**3082.0**	3083.1
rs16976728	3059.9	**3059.2**	3068.8	3068.5

QIC: Quasi-likelihood under the Independence model Criterion; SNP: single nucleotide polymorphism.
Figures in bold refer to smallest QIC value for each SNP (i.e., representing the best fitting genetic model).

Table S3. Association between genotypes of *IL4R* rs3024676 SNP and serum levels of IL-4 cytokine: results from IOW cohort study

**Genotypes of *IL4R* rs3024676**	**Serum levels of IL-4 (pg/mL)**	
	**n**	**median**
AA	5	0.06
AC	77	0.10
CC	180	0.07
	P value	0.10

Table S4. Association between genotypes of *IL4R* rs3024676 SNP and DNA methylation of *IL4R* gene: results from IOW cohort study

**Genotypes of *IL4R* rs3024676**	**DNA-methylation of CpG site cg26937798 (*M*-values)**	
	n	Median
AA	8	-3.78
AC	95	-3.87
CC	243	-3.58
	P value	0.001

Table S5. Information related to genotyped *IL4R* SNPs in the IOW cohort study

SNP	Sequence	Coordinate on Chromosome 16	Sequence Orientation	Assay Design ID	Accession Number
rs2057768	CTAGATGGGGGAACAGAGGTTTTACTATCTTGGTGCCTTTGGACCTGCTCCCAGGACTGA [T/C] GGAGCAGTCACAGATAAAGTCTGGGCATCTTGGGCAGGAACTTGAACAGTGGGACTGTAT	27229595	REVERSE	1616889629	NM_000418.2
rs6498012	TTAACCACGGTTCTGAGCTATCCAGCCCAAAGAGGAGAGTTTAAAAGGATTACATTTCTT [C/G] GTTGGAAATCAGTGAGCATAGTCGCCCAGAACCATTTTTGAAAAACTCAGAGAAGGCCTA	27239474	FORWARD	1616890550	NM_000418.2
rs1110470	GCAAGGGGGAGAAGGACTGGCTGGGATGGCAGCTGGAAGGTTGGCAGGCCAGGGACAACA [T/C] CGTCTGCCAAGCCATGGCAGTAGACTCAAACTCTGTCTCTAGGGCAGTGGGGA	27243927	FORWARD	1616888996	NM_001008699.1
rs3024604	AAACATAAAAAGAAGCAGAGAACACATACACATCTGCATCTTCCCTTGTTTACTTAACAA [T/C] AGATCTTGGAAGTCACTTCTCAGTAGAGGCTAGGTTGGGCAGAGCATTGGATTCTAGGCC	27270730	FORWARD	1616889945	NM_001008699.1
rs4787423	GGGTGGCAGGGACTTGCCCCTCTAGTCTGCCCCTTTGCAGTCCTCTCAGTCAATAATACG [T/C] ATTTACTGAGCAGCTACTACACACCTTGAGAGTAGAGCTGAGAACATATCGACAAGGACC	27274834	FORWARD	1616890360	NM_000418.2
rs3024676	AGGCTGGGCTTTGAAGAATGAATAGGAGTTTTTCAAGTGTCGAAACTGAACCCTGACCAA [A/C] CTTTGCTTTTGCAGACACTGGAAGAATTGTCTTACCAAGCTCTTGCCCTGTTTTCTGGAG	27281058	FORWARD	1616889962	NM_000418.2
rs1805011	CCATTCTCCTCTCCGAGCAGGTCCAGGAACAGGCTCTCTGTTAGCCGGGCCACAATGCCC [T/G] CCCTTCCCTCCTGGAAGTCATCCCTGCTGCTCTCAGGCGATGCACAGAAGCTCCCTTTTT	27281372	REVERSE	1700560211	NM_000418.2
rs1805012	TTCCTGGACCTGCTCGGAGAGGAGAATGGGGGCTTTTGCCAGCAGGACATGGGGGAGTCA [T/C] GCCTTCTTCCACCTTCGGGAAGTACGAGTGCTCACATGCCCTGGGATGAGTTCCCAAGTG	27281464	FORWARD	1617139833	NM_000418.2
rs8832	AATCCCGGCTGTCAAGGGGTGTTCAGTTAAGGGGAGCAACAGAGGACATGAAAAATTGCT [A/G] TGACTAAAGCAGGGACAATTTGCTGCCAAACACCCATGCCCAGCTGTATGGCTGGGGGCT	27283287	FORWARD	1612611002	NM_000418.2
rs3024685	CTCCAGTTTAAATAAACCTCTGACAAAAGGGTGAGTTATTCAACAGATTACCAGCATGAG [T/C] AACTGATGCTTACCTGCCGGGGATCTCTGGAAGACCATGCATGGCACATGCCCAGTTATG	27284410	FORWARD	1700560252	NM_000418.2
rs12102586	ATAGTTATCTGTTTAGGGCCAAGCAATATGCTAAGTGCCGTCCAGCCACTGTGTCATTTA [T/C] GTCTCCAAACAGCTCTAGTTGGGAGGCTCAATGATTATCCCAATTTTACAGATAAGGAAA	27285553	FORWARD	1616889172	NM_000418.2
rs16976728	TCTCCTTCCTAATCAAAAAGCAGCATGGAGGAGAAGGCTCCTTTGCCACCTTCCTTTCTG [T/C] TTTGGATGGTGCTTTTGCAATCATGAGGAGACAAGCCTGGAGATGAAAGGAGGCATTCTG	27289212	FORWARD	1700560196	NM_000418.2

(A) Linkage disequilibrium using *D*' values

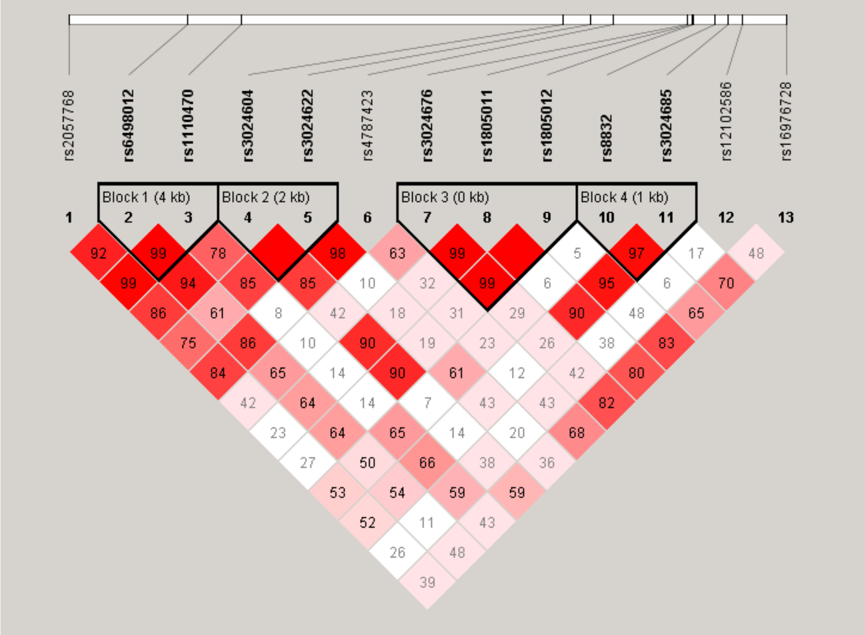

(B) Linkage disequilibrium using r^2 values

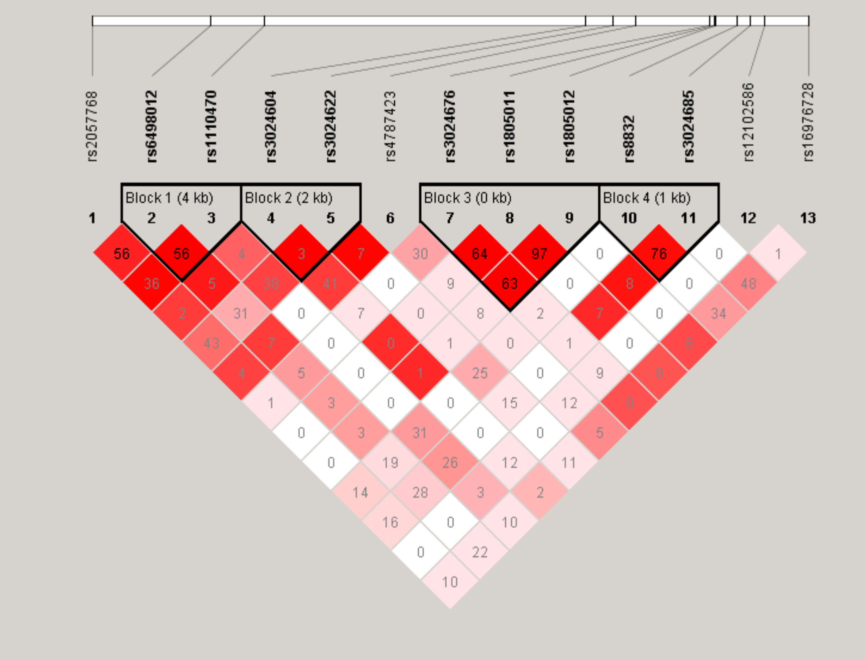

Figure S1. Estimates of linkage disequilibrium (LD) between *IL4R* single nucleotide polymorphisms. (A) LD estimates using D' values and (B) LD estimates using r^2 values.

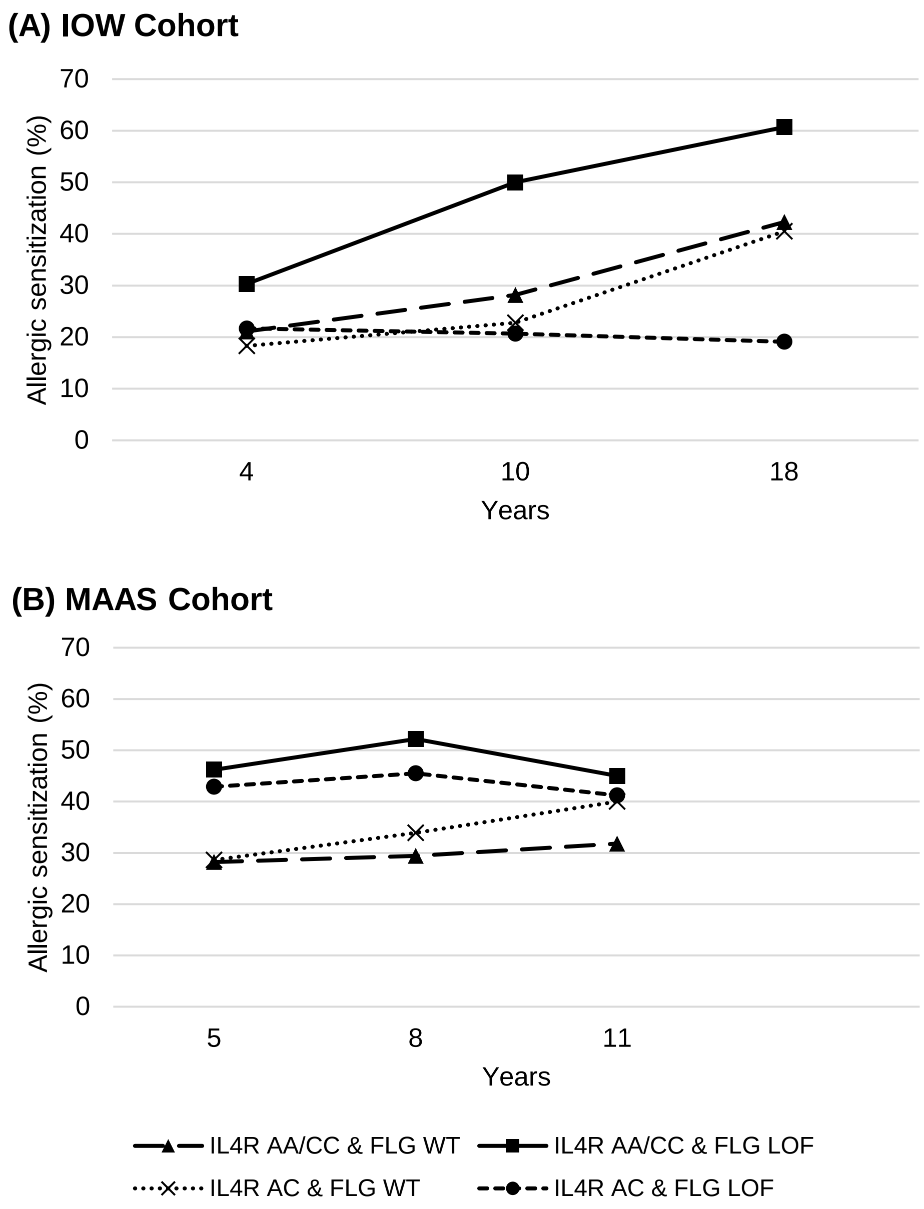

Figure S2. Proportion of allergic sensitization stratified by *FLG* and *IL4R* rs3024676 genotypes in the **(A)** IOW cohort and **(B)** MAAS cohort across different ages.
